# Calibrated meta-analysis to estimate the efficacy of mental health treatments in target populations: an application to paliperidone trials for treatment of schizophrenia

**DOI:** 10.1186/s12874-023-01958-w

**Published:** 2023-06-26

**Authors:** Hwanhee Hong, Lu Liu, Ramin Mojtabai, Elizabeth A. Stuart

**Affiliations:** 1 Department of Biostatistics and Bioinformatics, School of Medicine, Duke University, 2424 Erwin Road, Ste 1105, Durham, NC 27705 USA; 2 Department of Mental Health, Bloomberg School of Public Health, Johns Hopkins University, 615 N. Wolfe Street, Baltimore, MD 21205 USA

**Keywords:** Schizophrenia, Paliperidone Palmitate, Target Population, Individual-Level Data, Meta-Analysis, Generalizability, Randomized trials

## Abstract

**Backgrounds:**

Meta-analyses can be a powerful tool but need to calibrate potential unrepresentativeness of the included trials to a target population. Estimating target population average treatment effects (TATE) in meta-analyses is important to understand how treatments perform in well-defined target populations. This study estimated TATE of paliperidone palmitate in patients with schizophrenia using meta-analysis with individual patient trial data and target population data.

**Methods:**

We conducted a meta-analysis with data from four randomized clinical trials and target population data from the Clinical Antipsychotic Trials of Intervention Effectiveness (CATIE) study. Efficacy was measured using the Positive and Negative Syndrome Scale (PANSS). Weights to equate the trial participants and target population were calculated by comparing baseline characteristics between the trials and CATIE. A calibrated weighted meta-analysis with random effects was performed to estimate the TATE of paliperidone compared to placebo.

**Results:**

A total of 1,738 patients were included in the meta-analysis along with 1,458 patients in CATIE. After weighting, the covariate distributions of the trial participants and target population were similar. Compared to placebo, paliperidone palmitate was associated with a significant reduction of the PANSS total score under both unweighted (mean difference 9.07 [4.43, 13.71]) and calibrated weighted (mean difference 6.15 [2.22, 10.08]) meta-analysis.

**Conclusions:**

The effect of paliperidone palmitate compared with placebo is slightly smaller in the target population than that estimated directly from the unweighted meta-analysis. Representativeness of samples of trials included in a meta-analysis to a target population should be assessed and incorporated properly to obtain the most reliable evidence of treatment effects in target populations.

**Supplementary Information:**

The online version contains supplementary material available at 10.1186/s12874-023-01958-w.

## Introduction

Randomized controlled trials (RCTs) and meta-analyses based on these RCTs are the cornerstone of evidence-based clinical practice. While attention to internal validity of this evidence is critical, there is growing concern regarding a lack of external validity due to significant differences between the composition of participants of RCTs and the target populations [1–7]. Concerns about adequate diversity of the participants in RCTs have also increased attention to the composition of RCT samples and their representativeness. However, due to strict eligibility criteria applied in RCTs, RCT samples tend to exclude patients with mild (or severe) symptoms or comorbid disorders. This could make the RCT samples different from the target population [8]. In specific, psychiatric RCTs suffer from the unrepresentative participants issue mainly due to extremely heterogeneous psychiatric patients [9–11].

Disseminating and implementing findings of RCTs to a target population for routine care is important for clinical practice guidelines, cost-effectiveness research, and health-policy decision making. This population-based inference requires understanding generalizability of RCTs and correct estimation of an average treatment effect in the target population, denoted by *target population average treatment effect (TATE) *[12, 13]. However, current study designs and analysis approaches do not necessarily imply that existing RCT results generalize to relevant target populations [13].

Meta-analysis is often viewed as a way to approach the “true” effect of a treatment of interest because it combines information from multiple studies [14]. However, although the pooled sample in meta-analysis is often (implicitly) assumed to be more representative of the target population, even meta-analysis cannot guarantee accurate estimation of a TATE [15–18], especially when the (pooled) RCT sample does not represent patients in routine care settings with respect to characteristics that may modify treatment effects, resulting in effect heterogeneity [19]. Recently, two meta-analyses studying the efficacy of paliperidone in schizophrenia patients were published [20, 21], but they did not consider estimating TATEs in their meta-analyses.

**Fig. 1:**
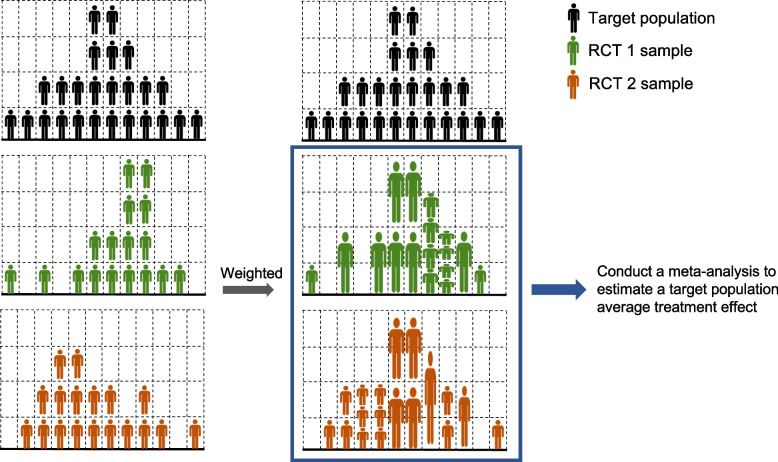
Illustration of the calibrated meta-analysis with hypothetical target population and samples of two RCTs. The black human icons represent sample of the target population and green and orange icons represent the sample of the two RCTs. The size of icon represents weights for each icon

With the growing trend in availability and use of individual-participant data (IPD) and availability of administrative data on target patient populations in usual care settings, it is now possible to take the composition of RCT samples into account and to adjust for their deviation from the target population. The estimates of TATEs in meta-analyses using IPD can be made more generalizable by strategically utilizing external target population data and assessing and adjusting the level of representativeness of RCTs [22, 23]. In particular, by using weighting methods to combine data from multiple RCTs, of which each represents slightly different parts of the overall population, more accurate population average treatment effects can be obtained (Fig. 1).

In this paper, we conducted such a calibrated meta-analysis using IPD from four paliperidone RCTs and with a external data source on a well-characterized target population sample. These RCTs studied the efficacy of paliperidone palmitate [24], a long-acting injectable atypical antipsychotic medication, compared to placebo, in treatment of individuals with schizophrenia. The goal of our study was to estimate the target population average treatment effect (TATE) of paliperidone palmitate in the treatment of schizophrenia among individuals with schizophrenia in usual care settings, rather than to examine the efficacy of paliperidone palmitate. The target population data came from a large sample of adults suffering from schizophrenia in the United States drawn from a pragmatic trial that aimed to recruit patients from a wide range of usual care settings.

## Methods

### Data collection and eligibility criteria

For the meta-analysis, eligible studies were phase III double-blind RCTs that studied the efficacy of paliperidone palmitate compared to placebo for treatment of schizophrenia and in which IPD were available. We searched trials in the Yale University Open Data Access (YODA) Project [25] and identified 5 RCTs available as of November 2015 with the following NCT IDs, NCT00074477 [26], NCT00111189 [27], NCT00210548 [28], NCT00101634 [29], and NCT00590577 [30]. All the RCTs were acute-phase trials, except NCT00111189 which was a relapse prevention trial. As such, we excluded NCT00111189 and a total of 4 RCTs were included in our meta-analyses. As of January 2022, one more eligible paliperidone palmitate RCT was identified, but it was not included in this analysis because data analyses had already been completed. The key eligibility criteria for participation in each of the 4 RCTs are presented in Table S1. All included RCTs randomized patients after a 7-day screening/washout period and followed them for 9 or 13 weeks.

We defined the *target population* as adults suffering from schizophrenia in usual care settings in the United States. To obtain data on the target population (individuals with schizophrenia in usual care settings) we used patients in the Clinical Antipsychotic Trials of Intervention Effectiveness (CATIE) study [31]. CATIE was a pragmatic trial supported by the National Institute of Mental Health to compare the effectiveness of antipsychotic drugs for treatment of schizophrenia among adults in usual care settings in the United States. CATIE aimed to enroll a broad sample of individuals with schizophrenia at 57 clinical sites by placing a premium on demographic and geographic diversity and employing few exclusion criteria (Table S1). For the analyses discussed here, we only used baseline characteristics from CATIE.

### Outcome measures and baseline covariates

Schizophrenia symptoms were measured based on the Positive and Negative Syndrome Scale (PANSS) total score, the sum of 30 items of which each ranges from 1 (absent) to 7 (extreme psychopathology) to assess various symptoms of schizophrenia [32]. The PANSS total score ranges from 30 to 210; higher scores indicate more severe symptoms. The primary efficacy outcome was the change in the PANSS total score between baseline and endpoint. A large PANSS reduction indicates greater improvement in schizophrenia symptoms between the beginning and end of the study. Baseline was defined as the first day of randomization. Endpoint was defined as the end of study (either 9 or 13 weeks) or the last-observation-carried-forward if the individual was lost from the study before the end of trial, as defined in the statistical analysis plans of the 4 RCTs. To account for varying follow-up time across the RCTs, we considered one secondary efficacy outcome: the PANSS total score change between baseline and Week 9, the shortest follow-up duration across all RCTs (Table S1).

We considered a total of 6 baseline covariates that were reported in the 4 RCTs and CATIE and could be potential effect modifiers: sex, race (white, African-American, other), age in years ($\le$ 30, 30–40, 40–50, and > 50), age in years at the first diagnosis of schizophrenia ($\le$ 20, 20–30, 30–40, and > 40), weight ($\le$ 70 kg, 70–80, 80–90, 90–100, 100–110, and > 110), and the PANSS total score.

### Statistical analysis

As illustrated in Fig. 1, the calibrated meta-analysis combined weighted RCT data. Patients in each RCT were weighted to equate the baseline characteristics between the RCT and the target population. As a result, the weighted RCT samples resemble the target population more closely than the unweighted samples.

More specifically, the calibrated meta-analysis involved three stages. First, trial participation weights were computed for all patients in each RCT. To calculate weights, for each RCT, we first formed a new dataset that stacked the data from the target population and that RCT. For each stacked dataset, we defined a population membership indicator as 1 for patients in the target population and 0 for patients in the RCT. Next, we fit a logistic regression of the membership indicator given the baseline covariates as predictors to estimate the probability of being in the target population for each RCT participant [33, 34]. These *participation scores* were denoted ${\widehat{e}}_{j}$, where $j$ indexes individuals. Next, the participation weights by the odds were defined as ${w}_{j}={\widehat{e}}_{j}/(1-{\widehat{e}}_{j})$ for the participants in each RCT [33–35]. Note that only participants in the trials were weighted to the target population. These weights were then used in subsequent analyses to make the RCT samples more similar to the target population on the baseline covariates. To assess this similarity, we calculated absolute standardized mean differences (ASMDs) of each of the baseline covariates between each of the RCTs and the target population [36]. We compared ASMDs calculated before and after weighting to assess how much the weighing improved similarity. In addition, we averaged the ASMDs of all baseline covariates for each RCT to quantify overall similarity for each RCT. An ASMD less than 0.1 is indicative of good balance in covariates between an RCT and the target population [37, 38]. Second, we estimated the TATE using each trial by fitting weighted regressions of the outcome with the weights ${w}_{j}$ using the survey package in R [39]. Third, we conducted a meta-analysis using the estimated TATEs. To account for between-study treatment effect heterogeneity, we fit a random-effects meta-analysis model with the DerSimonian and Laird inverse-variance method [40]. The standard deviation of the random effects, denoted by $\tau ,$ is used to assess the between-study treatment effect heterogeneity.

To obtain accurate TATE estimates, two key assumptions are required [19]. First, the span of the target population characteristics should be (at least somewhat) represented in RCTs, the so-called *positivity* assumption. This means that everyone in the population had to have a positive probability of participating in each RCT. Otherwise, we can only extrapolate results from the RCT to the represented part of the population. Second, there should be no unmeasured effect moderators. The participation weights can only adjust for differences in the observed baseline covariates (i.e., potential effect moderators) between each RCT and the target population. Unmeasured effect moderators may lead to unreliable TATE estimates.

We also carried out a random-effects meta-analysis using unweighted outcomes and compared the results. In addition, we conducted a subgroup analysis including only RCT patients from North America as a sensitive analysis. All analyses were executed using R version 3.6.3 [41].

## Results

A total of 1,738 patients were included in the meta-analysis (1,241 on paliperidone palmitate and 497 on placebo) along with 1,458 patients in CATIE. Table 1 presents baseline characteristics of participants in the paliperidone RCTs and CATIE. The RCTs included a slightly larger proportion of females (ranging from 30.7% to 33.7%) than CATIE (26.1%). The racial distributions across RCTs and between the RCTs and CATIE varied. The RCTs tended to have more participants who were neither White nor African-American. The distributions of age and onset age were comparable between RCTs and CATIE. CATIE included a higher proportion of patients with weight over 90 kg (42.2%) and a lower proportion of patients with weight less than or equal to 70 kg (18.3%) compared to the paliperidone RCTs (ranges are 16.1%—34.4% and 32.1%—46.3%, respectively). Compared to the CATIE participants, those in the paliperidone RCTs had a higher mean baseline PANSS score and a narrower range of baseline PANSS score, indicating more severe psychotic symptoms.

**Table 1:** Baseline characteristics for the CATIE and each of the RCTs. Proportions are presented for categorical variables and means and standard deviations in parenthesis are presented for continuous variables

	**Target Population**	**Randomized Controlled Trials**			
	**CATIE**	**NCT00074477**	**NCT00210548**	**NCT00101634**	**NCT00590577**
N	1458	243	349	511	635
Female (%)	26.1	32.9	30.7	33.7	32.8
Race (%)					
White	59.9	71.2	40.4	66.9	53.9
African-American	35.1	16.5	38.7	28.6	30.2
Other	5.0	12.3	20.9	4.5	15.9
Age, year (%)					
≤ 30	21.3	26.3	21.8	21.7	24.7
30–40	24.0	28.8	30.1	24.9	28.2
40–50	35.3	32.9	30.4	32.9	32.4
> 50	19.4	11.9	17.8	20.5	14.6
Onset age, year (%)					
≤ 20	30.2	31.7	35.1	31.6	32.9
20–30	46.2	41.2	43.1	40.7	43.1
30–40	16.1	20.4	16.1	18.1	19.4
> 40	7.5	6.7	5.7	9.6	4.6
Weight, kg (%)					
≤ 70	18.3	46.3	32.1	35.0	35.3
70–80	20.9	19.0	17.8	22.5	26.5
80–90	18.5	18.6	15.8	15.9	14.0
90–100	16.0	10.7	13.2	9.4	13.1
100–110	12.5	3.3	7.7	7.0	3.6
> 110	13.7	2.1	13.5	10.2	7.6
PANSS (mean [SD], [min, max])	75.65 (17.57) [31, 140]	87.86 (12.20) [55, 120]	91.09 (11.80) [70, 120]	90.85 (12.03) [70, 120]	87.07 (11.20) [61, 131]

**Fig. 2:**
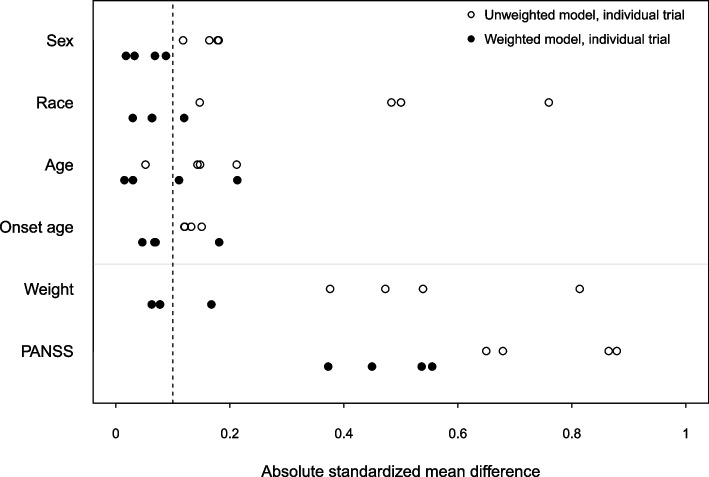
Absolute standardized mean differences of baseline covariates for each trial before weighting (hollow circle) and after weighting (solid circle) for each of the 4 paliperidone RCTs

Figure 2 and Table S2 displays ASMDs of the baseline covariates. Before weighting RCT samples, the ASMDs of all covariates except age from the NCT00101634 were greater than 0.1, indicating that these RCTs did not represent CATIE with respect to the six covariates. Specifically, the distributions of race, weight, and PANSS were more different between most RCTs and CATIE than those of sex, age, and onset age. After weighting, most ASMDs were closer to 0.1, although some differences remained on the PANSS total score. Overall, NCT00590577 represented CATIE well after weighting with the smallest average ASMD across covariates of 0.1 among the RCTs.

**Fig. 3:**
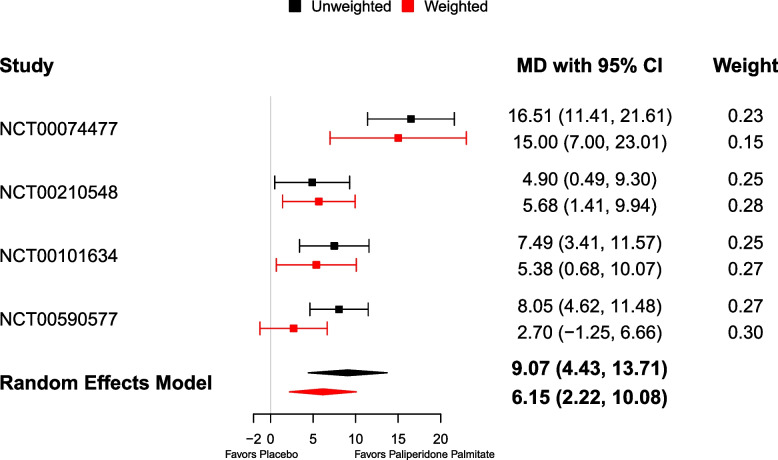
Forest plot for the mean difference of change in PANSS total score between paliperidone palmitate and placebo and 95% confidence intervals. Results from random effects meta-analysis models are plotted using diamond characters with the width indicating 95% confidence interval. The unweighted mean differences are plotted in black and the weighted mean differences are plotted in red

Figure 3 presents mean differences of change in PANSS total score between paliperidone palmitate and placebo before and after weighting. Before weighting, paliperidone palmitate appeared to be significantly more efficacious in reducing the PANSS total score compared to placebo. After weighting, however, except for one study (NCT00210548), the trial-specific TATEs became smaller. Meta-analyses showed significant effects under both unweighted (mean difference: 9.07 [95% CI: 4.43, 13.71]) and calibrated (6.15 [2.22, 10.08]) meta-analysis models, resulting in a smaller effect size for TATE. The estimated $\tau$ under unweighted and calibrated meta-analyses were 4.21 and 3.08, respectively, indicating moderate between-study heterogeneity.

Table S3 displays effect estimates from all secondary and sensitivity meta-analyses. The TATE estimates became smaller when using the endpoint measured at Week 9 (7.62 [2.37, 12.86]). When limiting the RCT samples to patients residing in North America, a total of 926 patients (53%) were included and the TATE estimate was smaller than the primary estimate (3.27 [0.15, 6.39] vs. 6.15 [2.22, 10.08]).

## Discussion

Generalizing results from meta-analysis of RCTs to a target population is not guaranteed without thorough assessment of the representativeness of RCTs included in the meta-analysis followed by proper calibration of the RCT samples. We introduced a calibrated meta-analysis approach to estimate target population average treatment effects and applied it to a meta-analysis of RCTs comparing paliperidone palmitate and placebo for treating patients with schizophrenia. By weighting patients in the RCTs, we made the weighted RCT samples more similar to the target population, represented by the CATIE sample. Our results showed that paliperidone palmitate was significantly more effective in reducing the PANSS total score than placebo under both unweighted and calibrated meta-analyses. However, the estimated TATE of paliperidone palmitate in calibrated analysis was smaller than the effect estimated from the unweighted meta-analysis, though the 95% confidence intervals overlapped, yielding unchanged conclusions regarding the treatment efficacy.

Our results reproduced the results from recently published two meta-analyses [20, 21]. Kishi et al. [20] included 5 paliperidone RCTs of which 4 were the same as RCTs included in our meta-analysis. Hodkinson et al. [21] used individual patient-level data identified at YODA, resulting in their meta-analysis including 5 paliperidone RCTs, of which 4 were the same as RCTs included in our meta-analysis and one was the RCT excluded from our meta-analysis. Both Kishi et al. and Hodkinson et al. found similar results as those under our unweighted meta-analysis, with similar point and 95% interval estimates of the PANSS total score change outcome.

### Implications

Our findings support that RCT samples and a target population may differ substantially on covariates (potential effect moderators), which may result in the effect estimates from an unweighted meta-analysis to deviate from what would be seen in the population. That is, treatment effects in RCTs samples and the target population may differ when the distributions of effect moderators differ between the samples. If there is no effect heterogeneity (no effect moderation) then RCTs and meta-analyses of RCTs will yield accurate inferences about population effects, though this rarely happens in practice [42–44]. The calibrated meta-analysis approach presented here can provide a tool for adjusting for potential moderators and thus better estimating TATEs. Furthermore, such calibration can change the magnitude of the efficacy estimates from meta-analyses and may even change the direction of the effect if the RCT samples deviate from target samples on important moderators. Evaluation of representativeness and calibration against data from target population when possible should be added to quality measures of individual participant meta-analysis such as the PRISMA-IPD Statement [45] and similar guidelines for IPD meta-analyses [46].

Current practice guidelines for treatment of mental disorders, such as the recent practice guidelines for treatment of schizophrenia published by the American Psychiatric Association [47] are based on meta-analyses of primary randomized controlled trials (RCTs) examining efficacy of different medication treatments and psychotherapies. However, the samples of these RCTs often deviate significantly from the target population of patients receiving care in usual care settings. As such, the practice guideline recommendations may not accurately reflect the effect of treatments when implemented in the real world. This is probably one of the reasons why treatments found to be efficacious in RCTs do not produce the same effects when implemented in the usual care settings [48]. Clinical decisions based on data from uncalibrated studies may not be optimal. Calibration of the meta-analyses against samples drawn from the target populations can potentially reduce the discrepancy between RCT efficacy and real-world effectiveness of treatments by adjusting the meta-analysis result for deviations of the RCTs samples from target populations.

The standardized mean difference between each RCT and the target population was used to assess representativeness of the RCTs after weighting. The imbalance between RCTs and CATIE samples was improved dramatically for most baseline features except PANSS and patient weight—likely because the RCT participants tended to have more severe symptoms than those in CATIE. The imbalance in weight may be related to national differences in average weight—the CATIE sample was drawn only from the United States; whereas the paliperidone RCTs also included participants from countries in Europe and Asia. Although the distributions of PANSS and weight between RCTs and CATIE were not perfectly similar in unweighted comparisons, there was considerable overlap in the distributions between the RCTs and CATIE, which meant that weighting could successfully improve the covariate balance. If there is no or little overlap of the distributions of effect moderators between the RCTs and target population (i.e., violation of the positivity assumption), statistical approaches may not estimate population treatment effects well because of the inherent extrapolation that will be required. The positivity violation cannot be formally tested, but it can be assessed by comparing distributions of baseline characteristics between RCTs and CATIE.

The weighting method used in the calibrated meta-analysis resulted in more similar samples between CATIE and RCTs, though it did not achieve fully equivalent samples. This may be due to some of the exclusion criteria applied to RCTs (i.e., RCTs exclude patients with comorbidities), and statistical analyses cannot solve fundamentally large differences between groups—if the trials really do not represent the target population then statistical methods cannot fully help. That said, the weighting approach helps make the combination of trials as similar to the population as possible, and so even though the distributions are not fully similar to CATIE the calibrated meta-analysis results do better reflect what we expect the TATE would be in the CATIE population.

### Limitations

This analysis has several limitations. First, we chose CATIE to represent the target population because it was among the best sources for providing data on the target population of patients receiving treatment for schizophrenia in a wide range of usual care settings in the United States. Furthermore, CATIE collected a broad range of demographic and clinical data from a large sample of diverse participants. However, target populations for future studies may be drawn from administrative data sources such as electronic health records or claims data and the results may differ by the choice of target population. This is a challenge of the calibrated meta-analysis as it depends on availability of truly representative target population samples along with individual patient RCT data—and consistent measurement of baseline characteristics between the two. Note that we had to use the coarsest category for age, age at onset, and weight variables in our analysis because several RCTs included in our meta-analysis did not have consistent continuous measures for those variables. Even when such data do exist, gaining access to the data remains a challenge. We hope that the current emerging movement of data sharing and data harmonization can resolve this challenge [49, 50].

Second, the participation scores and weights help balance the paliperidone RCTs and CATIE with respect to the observed covariates, but are not able to adjust for potential unobserved moderators. This is related to our results about the outstanding differences in treatment effect estimates between the unweighted and weighted analyses in NCT00590577. Knowing that the difference between NCT00590577 and CATIE is comparable with the differences between other RCTs and CATIE, this finding might be due to unobserved covariates that should have been adjusted for. When estimating TATEs is of primary interest, it is important to understand potential effect moderators in advance and collect the relevant information systematically and consistently across RCTs and target population data sources. In addition, further methodological research is required to handle unobserved effect moderators in calibrated meta-analysis.

### Future directions

In this study, we sought relatively large RCTs of a single medication treatment of schizophrenia conducted about the same time and using the same outcome measure (PANSS) as the CATIE trial (which represents the target population of interest). The identified paliperidone RCTs were conducted within a 5-year window (2003–2008), a time window that overlapped with CATIE’s timeframe (2000–2004). Focusing on a single medication and restricted time period reduces variations in outcomes and changes in population composition due to these factors. In addition, the methods examined could easily be used in other application areas – we are using the schizophrenia context here as a motivating example. Our methods can be used to examine generalizability for other disease conditions and treatments as well.

This paper focuses on how the TATE results are interpreted and implemented in clinical practice using a simple but widely-accepted method. However, multiple weighting methods are available, including flexible models such as generalized boosted model [51] and, Bayesian additive regression trees [33], and targeted maximum likelihood estimation [52] in calibrated meta-analyses. A subsequent paper that considered and compared those methods is currently under revision. Further method development and comparisons of multiple methods will be required to provide a practical guideline for method selection.

## Conclusion

Representativeness of samples of trials included in a meta-analysis to a target population should be assessed and incorporated properly to obtain the most reliable evidence of treatment effects in target populations. Calibrated meta-analysis, which integrates RCTs and population data, can be a powerful technique to estimate target population average treatment effects and draw population-level inferences. We recommend that when external data from target populations are available, these data be used to calibrate RCT samples and that future IPD meta-analyses be based on these calibrated RCT data.

## Supplementary Information

**Additional file 1: Table S1.** Key eligibility criteria and references for included studies. **Table S2.** Absolute Standardized mean differences of baseline covariates between each RCT and CATIE before weighting and after weighting in parentheses. **Table S3.** Mean difference of change in PANSS total score between paliperidone palmitate and placebo and 95% confidence intervals from all meta-analyses including secondary and sensitivity ones. 

## Data Availability

The paliperidone palmitate RCT data are accessible with an approval by the Yale University Open Data Access (YODA) Project and the CATIE study data are accessible with an approval for National Database for Clinical Trials from by the National Institute of Mental Health (NIMH). Individual investigators may reach out directly to YODA and NIMH to apply for those data access approvals. The authors are unable to share these data with individuals outside of the designated research team members.
