# Supplementary material for: Calibrated meta-analysis to estimate the efficacy of mental health treatments in target populations: an application to paliperidone trials for treatment of schizophrenia
Source: BMC Med Res Methodol. 2023 Jun 26;23:150. doi: 10.1186/s12874-023-01958-w (PMC10294408; doi:10.1186/s12874-023-01958-w)
Supplement: Supplementary file 1 — Additional file 1: Table S1. Key eligibility criteria and references for included studies. Table S2. Absolute Standardized mean differences of baseline covariates between each RCT and CATIE before weighting and after weighting in parentheses. Table S3. Mean difference of change in PANSS total score between paliperidone palmitate and placebo and 95% confidence intervals from all meta-analyses including secondary and sensitivity ones. [file 12874_2023_1958_MOESM1_ESM.docx]

**Supplementary Materials**

**Table S1**. Key eligibility criteria and references for included studies

**Table S2**. Absolute standardized mean differences of baseline covariates between each RCT and CATIE before weighting and after weighting in parentheses. The average of standardized mean differences across all covariates is reported.

**Table S3**. Mean difference of change in PANSS total score between paliperidone palmitate and placebo and 95% confidence intervals from all meta-analyses including secondary and sensitivity ones.

**Table S1**. Key eligibility criteria and references for included studies

|  | **CATIE** [1] | **NCT00074477** [2] | **NCT00210548** [3] | **NCT00101634** [4] | **NCT00590577** [5] |
| --- | --- | --- | --- | --- | --- |
|  |  |  |  |  |  |
| Follow-up | 23 months | 9 weeks | 13 weeks | 13 weeks | 13 weeks |
| Phase | Pragmatic RCT | Phase 2/3 | Phase 3 | Phase 3 | Phase 3 |
| Double-blind | Yes | Yes | Yes | Yes | Yes |
| Study start date | December 2000 | October 2003 | April 2005 | December 2004 | March 2007 |
| Study completion date | December 2004 | July 2004 | June 2006 | March 2006 | March 2008 |
| Countries | USA | International | International | International | International |
| Age | 18-65 | 18-65 | $\geq$18 | $\geq$18 | $\geq$18 |
| Sex | All | All | All | All | All |
| Diagnosis according to DSM-IV | DSM-IV criteria for  Schizophrenia; excluded first episode of schizophrenia^†^ | Diagnostic criteria for schizophrenia according to DSM-IV (295.10, 295.20, 295.30, 295.60, 295.90) for at least 1 year before screening | | | |
| PANSS score criteria | None | 70-120 at screening; 60-120 at baseline | 70-120 at screening and baseline | 70-120 at screening and baseline | 70-120 at screening; 60-120 at baseline |
| BMI (kg/m^2^) | None | 15-35 | >17 | >15 | >17 |
| DSM-IV Axis I other than schizophrenia |  | Excluded | Excluded | Excluded | Excluded |
| DSM-IV substance dependence within 3 months before screening |  | Excluded | Excluded | Excluded | Excluded |

^†^First episode of schizophrenia is defined if patients first began antipsychotic drug treatment for psychosis within the previous 12 months and have had psychotic symptoms for less than 3 years.

**Table S2**. Absolute Standardized mean differences of baseline covariates between each RCT and CATIE before weighting and after weighting in parentheses. The average of absolute standardized mean differences across all covariates is reported.

|  | **NCT00074477** | **NCT00210548** | **NCT00101634** | **NCT00590577** |
| --- | --- | --- | --- | --- |
| Female | 0.18 (0.09) | 0.12 (0.02) | 0.18 (0.07) | 0.16 (0.03) |
| Race | 0.48 (0.03) | 0.76 (0.06) | 0.15 (0.12) | 0.50 (0.06) |
| Age | 0.21 (0.21) | 0.14 (0.11) | 0.05 (0.03) | 0.15 (0.02) |
| Onset age | 0.13 (0.18) | 0.12 (0.07) | 0.12 (0.07) | 0.15 (0.05) |
| Weight | 0.81 (0.17) | 0.38 (0.08) | 0.47 (0.08) | 0.54 (0.06) |
| PANSS | 0.68 (0.55) | 0.88 (0.54) | 0.86 (0.45) | 0.65 (0.37) |
| Average of ASMDs | 0.42 (0.21) | 0.40 (0.15) | 0.31 (0.14) | 0.36 (0.10) |

**Table S3**. Mean difference of change in PANSS total score between paliperidone palmitate and placebo and 95% confidence intervals from all meta-analyses including secondary and sensitivity ones.

|  | Unweighted meta-analysis | Calibrated (weighted) meta-analysis |
| --- | --- | --- |
| ***Primary analysis*** | | |
|  | 9.07 (4.43, 13.71) | 6.15 (2.22, 10.08) |
| ***Secondary analysis*** | | |
| Endpoint at Week 9 | 7.62 (2.37,12.86) | 5.18 (1.45,8.91) |
| ***Sensitivity analysis*** | | |
| Including patients in North America only | 2.99 (0.56, 5.42) | 3.27 (0.15, 6.39) |

**References**

[1] Stroup TS, McEvoy JP, Swartz MS, Byerly MJ, Glick ID, Canive JM, et al. The National Institute of Mental Health Clinical Antipsychotic Trials of Intervention Effectiveness (CATIE) project: schizophrenia trial design and protocol development. Schizophrenia bulletin. 2003;29(1):15.

[2] Kramer M, Litman R, Hough D, Lane R, Lim P, Liu Y, et al. Paliperidone palmitate, a potential long-acting treatment for patients with schizophrenia. Results of a randomized, double-blind, placebo-controlled efficacy and safety study. The The International Journal of Neuropsychopharmacology. 2010;13(5):635-47.

[3] Gopal S, Hough DW, Xu H, Lull JM, Gassmann-Mayer C, Remmerie BM, et al. Efficacy and safety of paliperidone palmitate in adult patients with acutely symptomatic schizophrenia: a randomized, double-blind, placebo-controlled, dose-response study. International clinical psychopharmacology. 2010;25(5):247-56.

[4] Nasrallah HA, Gopal S, Gassmann-Mayer C, Quiroz JA, Lim P, Eerdekens M, et al. A controlled, evidence-based trial of paliperidone palmitate, a long-acting injectable antipsychotic, in schizophrenia. Neuropsychopharmacology. 2010;35(10):2072-82.

[5] Pandina GJ, Lindenmayer J-P, Lull J, Lim P, Gopal S, Herben V, et al. A randomized, placebo-controlled study to assess the efficacy and safety of 3 doses of paliperidone palmitate in adults with acutely exacerbated schizophrenia. Journal of clinical psychopharmacology. 2010;30(3):235-44.
